# Rural-Urban Differences in Survival and Nursing Home Use Among Medicare Beneficiaries Diagnosed With Dementia

**DOI:** 10.1093/geroni/igaa057.140

**Published:** 2020-12-16

**Authors:** Momotazur Rahman, Elizabeth White, Kali Thomas, Eric Jutkowitz

**Affiliations:** 1 Brown University School of Public Health, Providence, Rhode Island, United States; 2 Brown University, Providence, Rhode Island, United States

## Abstract

There is poor understanding as to how survival and healthcare utilization vary among older adults living with Alzheimer’s disease and related dementias (ADRD) in rural versus urban areas of the United States. This prospective cohort study used 2008-2015 Medicare claims linked with nursing home and home health assessment data to describe differences in survival and healthcare utilization in the six years following a new ADRD diagnosis between rural and urban populations. The sample consisted of 1,203,897 Medicare fee-for-service beneficiaries who were diagnosed with ADRD in 2008 or 2009. 77% (n=921,853) resided in metropolitan counties, 14% (n=162,857) in micropolitan counties, and 10% (n=119,187) in rural counties. Rural residents were on average about six months younger than metropolitan residents at diagnosis. Metropolitan residents survived a mean of 1211 days after diagnosis. Adjusting for individual characteristics, beneficiaries in rural and micropolitan counties survived 29.2 fewer days (95% CI -34.0,-24.4) and 31.9 fewer days (95% CI -36.1,-27.7) than metropolitan residents, respectively. Compared to metropolitan residents, rural residents spent 59.8 more days (95% CI 56.7, 63.0) in nursing homes. We found similar patterns in nursing home use for micropolitan vs. metropolitan residents, though the magnitude of the differences was smaller. Differences between groups became more pronounced the greater the time from diagnosis. These findings demonstrate that urban-dwelling older adults with ADRD are significantly more likely to remain in the community and less likely to use nursing homes than individuals in rural and micropolitan counties, particularly in later disease stages.

